# Phthiocerol dimycocerosates promote access to the cytosol and intracellular burden of *Mycobacterium tuberculosis* in lymphatic endothelial cells

**DOI:** 10.1186/s12915-017-0471-6

**Published:** 2018-01-04

**Authors:** Thomas R. Lerner, Christophe J. Queval, Antony Fearns, Urska Repnik, Gareth Griffiths, Maximiliano G. Gutierrez

**Affiliations:** 1 The Francis Crick Institute, 1 Midland Road, London, NW1 1AT UK; 2 Department of Biosciences, University of Oslo, Blindernveien 31, 0371 Oslo, Norway

**Keywords:** *Mycobacterium tuberculosis*, Tuberculosis, Phthiocerol dimycocerosates, PDIM, Lymphatic endothelial cells, hLEC, Macrophages, Phagosome damage, Cytosol, Xenophagy

## Abstract

**Background:**

Phthiocerol dimycocerosates (PDIM), glycolipids found on the outer surface of virulent members of the *Mycobacterium tuberculosis* (Mtb) complex, are a major contributing factor to the pathogenesis of Mtb. Myelocytic cells, such as macrophages and dendritic cells, are the primary hosts for Mtb after infection and previous studies have shown multiple roles for PDIM in supporting Mtb in these cells. However, Mtb can infect other cell types. We previously showed that Mtb efficiently replicates in human lymphatic endothelial cells (hLECs) and that the hLEC cytosol acts as a reservoir for Mtb in humans. Here, we examined the role of PDIM in Mtb translocation to the cytosol in hLECs.

**Results:**

Analysis of a Mtb mutant unable to produce PDIM showed less co-localisation of bacteria with the membrane damage marker Galectin-8 (Gal8), indicating that PDIM strongly contribute to phagosomal membrane damage. Lack of this Mtb lipid also leads to a reduction in the proportion of Mtb co-localising with markers of macroautophagic removal of intracellular bacteria (xenophagy) such as ubiquitin, p62 and NDP52. hLEC imaging with transmission electron microscopy shows that Mtb mutants lacking PDIM are much less frequently localised in the cytosol, leading to a lower intracellular burden.

**Conclusions:**

PDIM is needed for the disruption of the phagosome membrane in hLEC, helping Mtb avoid the hydrolytic phagolysosomal milieu. It facilitates the translocation of Mtb into the cytosol, and the decreased intracellular burden of Mtb lacking PDIM indicates that the cytosol is the preferred replicative niche for Mtb in these cells. We hypothesise that pharmacological targeting of PDIM synthesis in Mtb would reduce the formation of a lymphatic reservoir of Mtb in humans.

## Background

Worldwide, tuberculosis (TB) is the leading cause of death from an infectious disease with over 1.3 million TB-related deaths in 2016 [1]. The etiological agent of TB, *Mycobacterium tuberculosis* (Mtb), has a complex and unique cell wall with several components on the outer surface that Mtb uses to interact with the host cells it infects [2, 3]. One such set of components, phthiocerol dimycocerosates (PDIMs), are methyl-branched fatty acid-containing lipids, which have been found on all pathogenic members of the Mtb complex [4] and on all clinical isolates of Mtb [5]. In contrast, PDIMs are known to be frequently lost during in vitro culture [5, 6]. This implicates PDIM as a major factor that contributes to the pathogenesis of TB. Indeed, Mtb mutants lacking PDIM are severely attenuated in a guinea pig TB model [5] (with reduced replication and fewer lung tubercles than seen with a PDIM-positive strain), as well as in a mouse TB model [7–9]. PDIMs have been implicated in several functions during Mtb infection, including but not limited to: phagocytic uptake of Mtb in human macrophages [10], reduced recruitment of vacuolar-type H^+^-ATPase (v-ATPase) and decreased phagosome-lysotracker association [10], protection from nitric oxide-dependent killing by macrophages [11], masking of pathogen-associated molecular patterns leading to reduced recruitment of microbicidal macrophages [12] and induction of host cell death [13]. Recently, PDIMs have been shown to act in concert with the ESAT-6 secretion system (ESX-1) to promote phagosomal rupture/damage in primary human macrophages [14], THP-1 macrophages [15] and murine bone marrow-derived macrophages [16]. Although these studies concluded that phagosomal membrane rupture is reduced in the absence of PDIM, without ultrastructural data there has been no unequivocal confirmation that this condition reduces the extent to which Mtb localises in the cytosol.

Myelocytic cells (such as macrophages and dendritic cells) are the primary hosts for Mtb after infection [17] and virtually all in vitro studies of PDIM biology have used murine or human macrophages as a model. However, Mtb can infect many other cell types [18]. We have previously shown that primary human lymphatic endothelial cells (hLECs) are a reservoir for Mtb in humans [19]. Lymphatic endothelial cells form part of the walls of lymph nodes and lymphatic vessels [20, 21] but are also increasingly recognised as having a key role in the innate and adaptive immune response to infection [22–26]. We showed in vitro that Mtb can be taken up by hLECs, inside which they disrupt the phagosomal membrane and translocate to the cytosol, a process involving the secretion of ESAT-6 by the type VII secretion system ESX-1, encoded within the RD-1 locus [27, 28]. We hypothesised that after escaping from the phagosome, Mtb replicates preferentially in the cytoplasm of hLECs, and we observed minimal host cell death throughout this process [19]. Given the potential importance of cytosolic localisation for efficient Mtb replication in hLECs, and the emerging role for PDIM in phagosome damage, we sought to determine if PDIM were important during Mtb infection of hLECs.

We used an in vitro model using primary hLECs infected with either wild-type (WT) Mtb or a mutant lacking the ability to produce PDIM. Although over 50 kb of the Mtb genome (>1%) is dedicated to producing PDIM [29, 30], mutation of any of several biosynthetic or transporter genes is sufficient to prevent completely PDIM production or its transfer to the outer surface [7, 8, 31, 32]. One such biosynthetic gene (*ppsE*, encoding phthiocerol synthesis polyketide synthase type I) was shown to be essential for PDIM production [33, 34]. In this study, we used Mtb H37Rv strain PMM100, which has an unmarked deletion of *ppsE* [10]. We also used the WT parent strain (Mtb WT) and the PMM100 mutant complemented with *ppsE* [10]. Each strain also expressed a chromosomally integrated green fluorescent protein (GFP) tag [10].

We show that PDIM strongly contributes to the process of phagosome damage in hLECs, and that lacking PDIM leads to a significantly increased proportion of Mtb present in phagolysosomes relative to WT Mtb. We also determined that PDIM contributes towards the induction of selective autophagy of the bacteria (xenophagy). Using transmission electron microscopy (TEM), we confirmed that lacking PDIM significantly reduces the number of bacteria present in the cytosol. Finally, we show that the burden of Mtb lacking PDIM is severely reduced in hLECs, in contrast to the unaffected bacterial burden seen in macrophages under similar infection conditions [10, 14]. We suggest that this defect is due to a fundamental difference in the primary replicative niche of Mtb in hLECs compared to macrophages – namely the cytosol – which is much less accessible when PDIMs are absent. Moreover, translocation to the cytosol may be a strategy that Mtb employs to avoid being killed in a phagolysosome.

This work identifies PDIM as an important factor for Mtb to access and translocate into the cytosol of lymphatic endothelial cells and thus, to a potentially replication-favourable environment. An important consequence of this scenario is its implication that pharmacologically targeting PDIM synthesis could severely inhibit the formation of a lymphatic reservoir of Mtb in humans.

## Results

### PDIM increases the ability of Mtb to damage the phagosome and avoid a phagolysosomal fate in hLECs

We infected hLECs with Mtb-GFP WT (Mtb WT), Mtb-GFP PMM100 (Mtb ΔPDIM) or Mtb-GFP PMM100::pMVE (Mtb ΔPDIM::PDIM) at a multiplicity of infection (MOI) of ten per cell for a period of 5 h prior to washing away any extracellular bacteria. We then allowed the infection to proceed for 2, 24, 48 or 72 h (5 + 2 h, 5 + 24 h etc.) before the samples were fixed and labelled with specific antibodies to identify different intracellular markers.

**Fig. 1:**
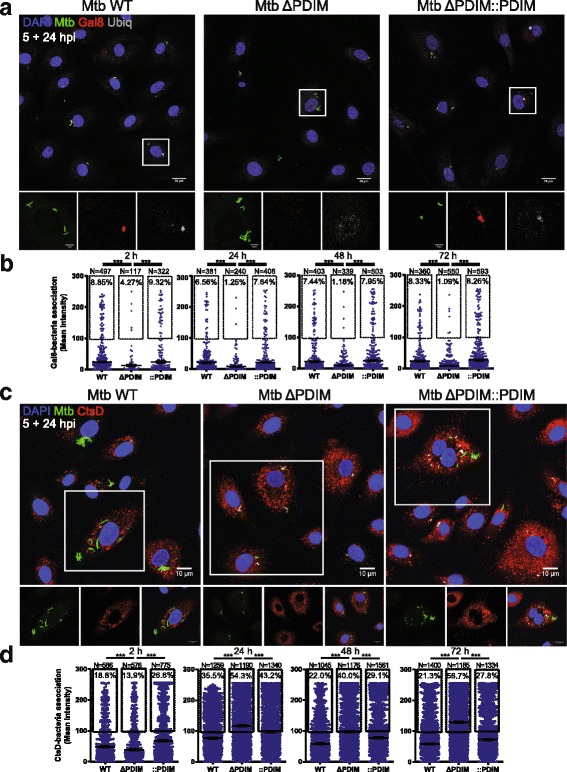
PDIMs contribute to phagosome damage and help avoid a phagolysosomal fate in hLECs, which were infected with Mtb WT, Mtb ΔPDIM or Mtb ΔPDIM::PDIM (all GFP tagged) for 5 h before any non-phagocytosed bacteria were washed away. The infection proceeded for 2, 24, 48 or 72 h before fixation. The samples were subject to immunofluorescence labelling using antibodies against (**a**) Galectin 8 (Gal8) or (**c**) Cathepsin D (CtsD). Nuclei were visualised with DAPI. White boxes outline the zoomed area below which the fluorescent channels are displayed separately. Scale bars are 10 μm. Images are representative examples from samples at 5 + 24 hpi. Images such as in (**a**) and (**c**) were quantified using ImageJ to measure the association of (**b**) Gal8 or (**d**) CtsD to each strain of Mtb at each time point. The percentage of Mtb that was positive for the marker [defined as the marker (the red channel) having a mean pixel intensity of at least 100 in the area overlapping with the individual Mtb particles (the green channel)] in each condition is shown. At least six fields of view were imaged per sample per replicate and three independent replicates were performed. The total number of analysed Mtb particles is also displayed for each condition (*N*). The overall mean is shown with error bars representing the standard error of the mean. Statistical significance was determined using one-way ANOVA with Tukey’s post-test. CtsD Cathepsin D, Gal8 Galectin 8, GFP green fluorescent protein, hLEC human lymphatic endothelial cell, hpi hours post-infection, Mtb *Mycobacterium tuberculosis*, PDIM phthiocerol dimycocerosate, WT wild type, *** *p* < 0.001

First, we investigated whether PDIMs were involved in phagosome damage in hLECs using the marker Galectin 8 (Gal8) [35, 36] (Fig. 1a,b). Using an unbiased image-based analysis (see ‘Methods’) to quantify the association of Gal8 to either bacterial strain, we determined that after 5 + 2 hpi, there was a 50% reduction in the extent to which Mtb in phagosomes was positive for this cytoplasmic marker when PDIM was absent (Fig. 1b). Complementation of the Mtb ΔPDIM strain with PDIM reverted the proportion of Gal8-positive bacteria to WT levels. The effect was more profound at later time points (24–72 h), when only 1–2% of Mtb phagosomes were Gal8-positive in the absence of PDIM, compared to 6–9% with PDIM present (Fig. 1b). We also measured the association of ubiquitin and other xenophagy markers using the same method (see below).

Phagosome damage is a prerequisite for localisation of Mtb in the cytosol, in a process that requires the type VII secretion system encoded by the RD-1 locus [27, 28]. We previously determined that Mtb lacking the RD-1 locus was over three times more likely than Mtb WT to be present in phagolysosomes in hLECs [as measured by Cathepsin D (CtsD) association] [19] and several studies have shown that PDIM-less mutants are transported to acidic compartments in macrophages [10, 37, 38]. We, therefore, reasoned that because the Mtb ΔPDIM strain was less capable of damaging the phagosomal membrane, it would be more frequently localised in phagolysosomes (Fig. 1c,d). Indeed, we found that after 5 + 24 hpi, there was a significant 1.5-fold increase in the proportion of Mtb co-localising with CtsD compared to Mtb WT. This increased to 1.8-fold at 5 + 48 h and 2.75-fold at 5 + 72 h (Fig. 1d). Thus, whereas PDIM increased the number of damaged Mtb WT phagosomes, the lack of this lipid retained Mtb in phagolysosomes.

### NF-kB and IRF-1 are not differentially activated in hLECs by Mtb lacking PDIM

**Fig. 2:**
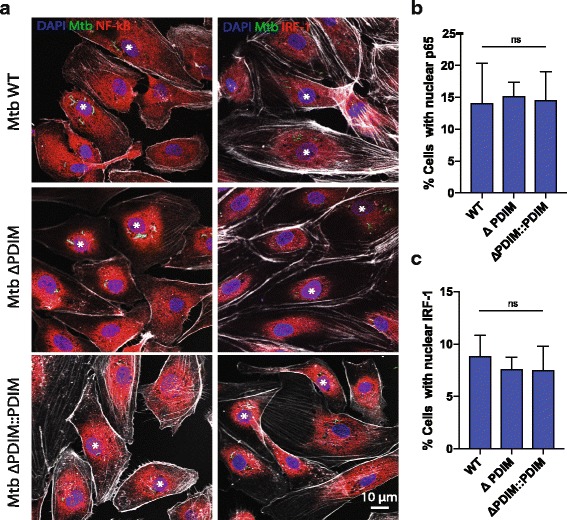
PDIMs do not differentially affect NF-kB or IRF-1 activation in human lymphatic endothelial cells, which were infected with Mtb WT, Mtb ΔPDIM or Mtb ΔPDIM::PDIM (all GFP tagged) for 5 h before any non-phagocytosed bacteria were washed away. The infection proceeded for 24 h before fixation. The samples were subject to immunofluorescence labelling using antibodies against p65 (NF-kB) or IRF-1. Nuclei were visualised with DAPI. **a** Representative images of infected cells. White asterisks show infected cells with nuclear translocation. Percentage of infected cells with NF-kB (**b**) or IRF-1 (**c**) nuclear localisation. At least 100 infected cells were counted and three independent replicates were performed. The mean is shown with error bars representing the standard error of the mean. Statistical significance was determined using one-way ANOVA with Tukey’s post-test. GFP green fluorescent protein, Mtb *Mycobacterium tuberculosis*, ns not significant, PDIM phthiocerol dimycocerosate, WT wild type

Mtb PDIMs are reported to mask Toll-like receptors (TLR) ligands [12] and TLR activation can impact phagosome maturation in macrophages [39]. Thus, we investigated whether the increased kinetic of Mtb targeting to phagolysosomes was due to the differential activation of hLECs by Mtb WT and mutants. We monitored the activation of NF-kB (p65) and IRF-1 after infection of hLECs with Mtb WT, Mtb ΔPDIM and Mtb ΔPDIM∷PDIM by confocal microscopy as previously shown [40, 41]. We found that after 24 h of infection, Mtb induced NF-kB activation, as measured by translocation of p65 into the nucleus but this activation was not significantly different between Mtb WT and the mutants (Fig. 2a,b). Additionally, Mtb induced IRF-1 activation, as measured by IRF-1 translocation into the nucleus (Fig. 2a). However, this activation was not significantly different between Mtb WT and the mutant lacking PDIM (Fig. 2c). Our results argue that, unlike macrophages, PDIM does not impact TLR activation in hLEC.

### Recruitment of xenophagy-related proteins is impaired when Mtb lacks PDIM in hLECs

Xenophagy is the attempted destruction of intracellular pathogens by (selective) autophagy [42]. It has been shown to be a defence mechanism by which Mtb proliferation is controlled by macrophages [43] and hLECs [19]. For Mtb, the process is thought to involve the recognition of the bacterium itself [44], the damaged membrane surrounding the bacterium [45] and/or Mtb-derived products such as extracellular DNA [46]. Ubiquitin (Ub) serves as an autophagy receptor, forming polyubiquitin chains on these targets and adaptor proteins such as sequestosome 1 (p62) and antigen nuclear dot 52 kDa protein (NDP52) form a bridge between the ubiquitin chain and a forming double membrane structure called a phagophore; the latter is positive for microtubule-associated proteins 1A/1B light chain 3B (LC3) [46]. Eventually the phagophore fuses to form a structure that completely encapsulates the bacterium; this is called an autophagosome.

**Fig. 3:**
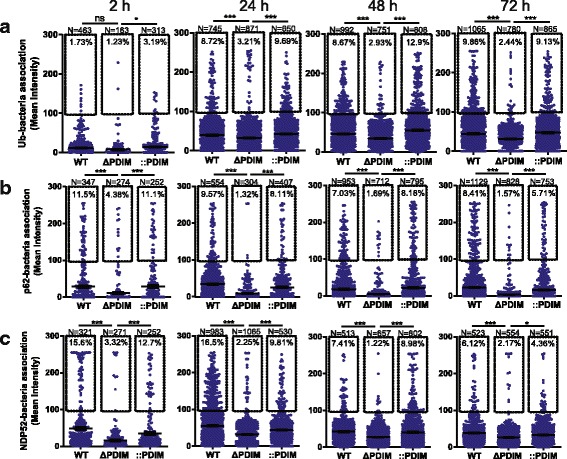
Recruitment of xenophagy markers to Mtb in human lymphatic endothelial cells is impaired when PDIMs are absent. Quantification of immunofluorescent analysis of three proteins involved in xenophagy: **a** ubiquitin (Ub), **b** p62 and **c** NDP52. The process of infection, immunofluorescent labelling, imaging and quantification was identical to that explained in Fig. 1. At least six fields of view were imaged per sample per replicate and three independent replicates were performed. The total number of analysed Mtb particles is also displayed for each condition (*N*). The overall mean is shown with error bars representing the standard error of the mean. Statistical significance was determined using one-way ANOVA with Tukey’s post-test. Mtb *Mycobacterium tuberculosis*, ns not significant, PDIM phthiocerol dimycocerosate, Ub ubiquitin, WT wild type, *** *p* < 0.001, * *p* < 0.05

We previously showed that Mtb lacking the RD-1 region was not efficiently targeted by autophagosomes in hLECs [19]. This was hypothesised to be due to the requirement for a damaged phagosome to allow recognition by the selective autophagy machinery of either Mtb or the damaged membrane itself. Therefore, given that Mtb lacking PDIM was significantly less associated with Gal8, we predicted that the mutant bacteria would be less frequently associated with components of the xenophagy pathway. Using immunofluorescence labelling, we measured the association of ubiquitin (Fig. 3a), p62 (Fig. 3b) and NDP52 (Fig. 3c) with WT and mutant Mtb at 2–72 hpi. There was initially a very low level of association of ubiquitin with Mtb WT at 5 + 2 hpi, but this increased to 8–10% at 5 + 24 hpi and remained at this level throughout (Fig. 3a). In contrast, from 24 to 72 hpi, Mtb ΔPDIM showed very low levels of ubiquitin when compared to the WT strain (63% at 24 h to 86% reduction at 72 h; Fig. 3a). In agreement with the role of PDIM in the recognition by ubiquitin, the PDIM complemented strain had levels comparable with the WT strain. When p62 (Fig. 3b) and NDP52 (Fig. 3c) localisation was analysed, similar results were obtained. Overall, 5–15% of the WT bacteria were associated with autophagic markers throughout the infection, which is consistent with other studies of macrophages [46]. Deletion of PDIM reduced these levels to 1–5%, indicating that PDIMs contribute towards recruitment of xenophagy proteins.

### Mtb lacking PDIM is much less frequently localised in the cytosol in hLEC

**Fig. 4:**
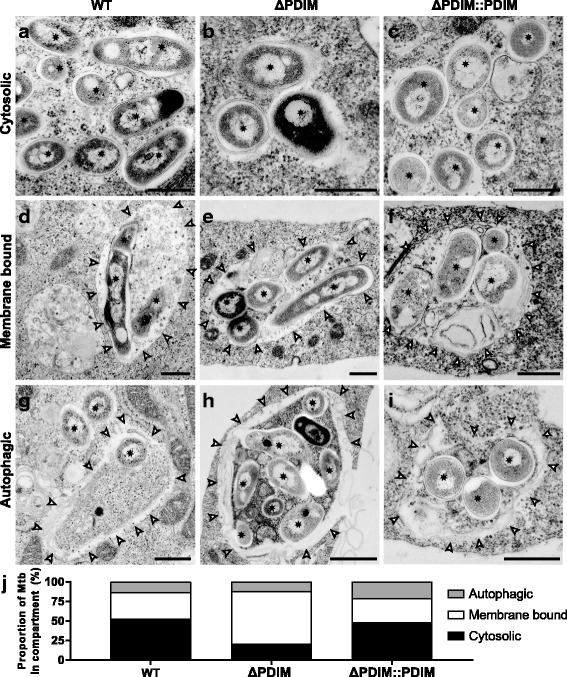
Electron microscopy analysis. Mtb lacking PDIM is much less frequently present in the cytosol. Human lymphatic endothelial cells were infected with Mtb WT, Mtb ΔPDIM or Mtb ΔPDIM::PDIM for 5 + 24 h and fixed. The samples were processed for resin embedding and then transmission electron microscopy was used to look at the subcellular localisation of Mtb. Three potential localisations were considered and classified. **a–c** Cytosolic (defined as no obvious host membrane surrounding bacteria outside the bacterial capsule-like material visible on the surface). **d**–**f** Phagosomal (defined as bacteria enclosed in a single membrane-bound organelle). Most of these are larger (membrane-bound) vacuoles, often with more than one Mtb cell as well as additional irregular membranes and contents in various stages of degradation, typical for phagolysosomes. Arrowheads indicate the phagosomal/phagolysosomal limiting membrane. **g**–**i** Autophagic. In this case, the bacteria and surrounding cytoplasm are enclosed by either single or multiple membranes that are mostly poorly preserved. In contrast to phagolysosomes, the inner contents of the autophagosomes do not appear to be degraded. The arrowheads here indicate the poorly visible membrane or remains of membranes on the periphery of the structure. In all images, black stars indicate bacteria. Scale bars are 500 nm. **j** Stereological analysis was performed to determine the proportion of Mtb in each compartment. Data are representative of two independent experiments with at least 32 infected cells analysed for each condition. Mtb *Mycobacterium tuberculosis*, PDIM phthiocerol dimycocerosate, WT wild type

Because the Mtb WT showed a higher frequency of phagosomal damage than Mtb ΔPDIM, the next prediction was that the WT strain would be more likely to be physically translocated to the cytoplasm. For this, we performed conventional resin embedding and TEM on hLECs infected with each strain for 24 h (Fig. 4a–i). We had previously established conditions in these cells in which Mtb in intact phagosomes could be clearly distinguished from those that had physically translocated to the cytoplasm [19]. Here, we also discriminated between Mtb in phagosomes/phagolysosomes and those surrounded by autophagic structures. The ultrastructural criteria used to distinguish the three different sub-populations are described in more detail in the legend of Fig. 4. After acquiring electron microscopy images, we used an unbiased stereology approach to quantify the proportion of bacteria that were localised to the three different compartments. The data showed that compared to Mtb WT, there was a 60.4% reduction in the proportion of Mtb ΔPDIM localised in the cytosol, with a corresponding increase in membrane-bound (but not autophagic) bacteria. Complementation of Mtb ΔPDIM with PDIM reverted the localisations back to WT levels. Therefore, we concluded that the presence of PDIM in Mtb WT facilitates the ability of the bacteria to escape from the special conditions within the phagosome and to be exposed to a different environment in the cytoplasm.

### Intracellular burden of Mtb lacking PDIM is severely reduced in hLECs

**Fig. 5:**
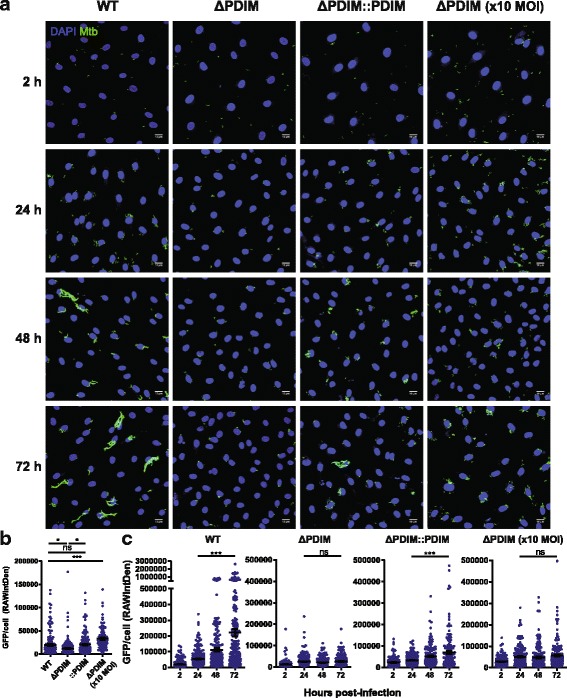
Intracellular burden of Mtb lacking PDIM is severely reduced in human lymphatic endothelial cells. **a** Representative images of human lymphatic endothelial cells infected with Mtb WT, Mtb ΔPDIM or Mtb ΔPDIM::PDIM at each time point. In addition, Mtb ΔPDIM was infected at 10 times the usual MOI to overcompensate for the reduced phagocytic uptake of this strain. **b** The phagocytic uptake for each strain was measured using ImageJ by plotting the total GFP signal (called the RAWIntDen) per cell at 5 + 2 hpi. At least six fields of view were analysed per replicate with three independent replicates performed. The overall average GFP per cell is shown with error bars representing the standard error of the mean. Differences between the mean for each strain were tested using one-way ANOVA with Tukey’s post-test. **c** The GFP per cell was measured in the same way as described in (**b**) except each time point for each strain is compared (i.e. for bacterial replication). GFP green fluorescent protein, hpi hours post-infection, MOI multiplicity of infection, Mtb *Mycobacterium tuberculosis*, ns not significant, PDIM phthiocerol dimycocerosate, WT wild type, *** *p* < 0.001, * *p* < 0.05

Although PDIMs have been shown to be essential for replication in vivo [5, 7–9], PDIM deficiency was shown not to affect Mtb burden in human macrophages under very similar conditions to those used in this study [10, 14]. However, there are some fundamental differences between hLECs and macrophages that suggest lacking PDIM would affect Mtb burden in hLECs. For instance, hLECs have a considerably higher proportion of Mtb localised in the cytosol than macrophages [19, 47]. Moreover, in contrast to the substantial amount of cell death observed in Mtb-infected macrophages, infection of hLECs with Mtb leads to minimal host cell death [19, 48, 49]. Since both strains of Mtb are labelled with GFP, we could use the total GFP signal per cell as an indicator of net bacterial growth. We, therefore, quantified the total GFP signal per hLEC cell (GFP/cell) from 2 to 72 hpi with each Mtb strain (Fig. 5a). Measurements of the colony-forming units were not used because of the inaccuracy deriving from the clumping of Mtb, as described previously [19]. Because PDIMs have been shown to be required for efficient phagocytosis in primary human macrophages [10], we first checked the uptake of each Mtb strain at 2 hpi (Fig. 5b). Consistent with previous results, there was on average a 38.2% reduction in uptake of Mtb ΔPDIM compared to the WT strain. To compensate for this reduction, we infected hLECs with Mtb ΔPDIM at a tenfold higher MOI, resulting in an average increase in uptake of 67.1% compared to the WT strain (Fig. 5b). We then measured the increase in GFP/cell signal over the course of 72 h of infection, and showed that replication of Mtb is severely inhibited when lacking PDIM in hLECs, with on average an 11.1-fold increase in WT compared to a 2.12-fold increase in the mutant (Fig. 5c). Even when overcompensating for the reduced uptake using a tenfold higher MOI, the average GFP/cell was much less than that for the WT strain with the normal MOI, and showed a similar 2.03-fold increase as seen for this strain with the normal MOI (Fig. 5c).

## Discussion

Some intracellular pathogens such as Mtb can damage phagosomal membranes [35]. When this happens, glycans present on the inner leaflet of intracellular membranes can be exposed to the cytosol and are recognised by cytosolic sensors such as galectins [35]. In turn, galectins recruit the selective autophagy machinery and thus, the pathogen is targeted by the autophagy pathway and eventually killed after autophagosome–lysosome fusion. This process is called xenophagy, which is the selective autophagy of a foreign object (in this case, Mtb). In this study, we used Gal8 as a marker of damaged phagosomes and in combination with autophagy markers (p62, Ubiquitin and NDP52), we examined the role of PDIM in the xenophagic recognition of Mtb in hLECs. Phagosome damage and xenophagy markers were significantly less associated with Mtb lacking PDIM than WT. The simplest explanation for this result is that phagosome damage is likely to be a prerequisite for exposure of Mtb itself, Mtb-derived components such as DNA and the host glycans on the inner leaflet of the phagosome membrane to the xenophagy machinery. Indeed, the higher fraction of Mtb WT-associated structures that label for the xenophagy markers is consistent with the higher percentage of phagosomes that are accessible to Gal8. Although the association of markers with Mtb is generally low (for example, 6–10% of Mtb WT and only 1–4% of Mtb ΔPDIM co-localise with Gal8 at each snapshot in time from 2–72 hpi; Fig. 1a,b), it is likely that events in phagosomal damage, pathogen recognition and targeting of autophagic compartments are dynamic and transient [36], but this remains to be determined precisely. Importantly, the differences in the intracellular trafficking of Mtb are likely independent of TLR activation since NF-kB and IRF-1 activation in hLECs was similar for Mtb WT and Mtb lacking PDIM.

Our data show that Mtb lacking PDIM more frequently co-localises with the lysosomal marker CtsD than Mtb WT (54% versus 35% at 24 hpi) (Fig. 1c,d). This differs from what has been reported for human macrophages, where Mtb with and without PDIM equally associates with the lysosomal marker CD63 (around 25% at 24 hpi) [10]. Whereas it is possible that the trafficking of Mtb in macrophages and hLECs is different, we hypothesise that this reduction is due to the ability of Mtb to localise in the cytosol of hLECs and thus, to avoid a phagolysosomal fate. Supporting this, we measured the effect of PDIM on the localisation of Mtb in the phagosome vs. the cytosol (Fig. 4) for the first time. We determined that approximately 50% of the bacteria successfully translocate to the cytosol after 24 h, but only 21% in the absence of PDIM (Fig. 4). The recent reports on the involvement of PDIM on phagosome damage [14–16] did not determine if the translocation of Mtb to the cytosol was affected because those techniques and experiments were unable to discern between cytosolic localisation and access. By using these methods, the formation of a single damaged phagosome will give a positive result without determining the extent of cytosolic localisation, a critical event that needs to be considered. Here, we used TEM, which, when the samples are carefully prepared, does show the presence or absence of host membranes around Mtb. Although chemical fixation and subsequent dehydration steps could affect the preservation of the phagosomal membrane and increase the total numbers of phagosomes with damaged membranes, the comparison between the different mutants unambiguously shows that PDIM contributes to the cytosolic translocation of Mtb in hLECs. This has yet to be seen in macrophages.

We hypothesised that in hLECs, Mtb replicates preferentially in the cytosol. Consistent with this, the PDIM mutant, which escapes less far into the cytosol, is significantly inhibited for growth in hLECs. Although PDIM mutants are attenuated in vivo [5, 7–9], to our knowledge in cellulo models of Mtb infection using PDIM mutants show that at high MOI, there is no replication defect of PDIM-less strains [10, 14]. Moreover, at low MOIs, PDIM-deficient strains show only a moderate replication defect [13, 14] or no significant difference [11]. One study using transposon mutants in PDIM loci shows that three of five strains have a reduced burden, whereas two of five are the same as WT; however, these differences may be due to off-target effects, which are possible when using transposons [8]. In this study, we used a high MOI to be consistent with the literature and in agreement with our previous studies [19]. We show that, unlike what is reported for macrophages, Mtb ΔPDIM has a significantly reduced burden compared to WT. Even infecting with a tenfold higher MOI (overcompensating for reduced uptake of ΔPDIM) does not lead to a higher burden than the WT at the original MOI. Therefore, there is a clear attenuation when PDIM is absent in hLECs.

The in vivo attenuation of PDIM mutants is thought to result from increased susceptibility to reactive nitrogen intermediates produced by macrophages as well as the enhanced activation of the immune response early during infection [11]. PDIM-less mutants have been shown to be less efficient at preventing v-ATPase recruitment to the phagosome membrane and are more frequently found in acidified compartments. This did not, however, lead to differences in the extent of Mtb in the phagolysosome [10], perhaps explaining why there was no difference in intracellular burden. In contrast, we observed a marked increase in the extent to which the PDIM mutant co-localised with the phagolysosome marker CtsD. This result was comparable to what we observed with a RD-1 mutant in the same model and with the same infection conditions. We previously showed that a large proportion of Mtb cells translocate into the cytosol in hLECs without causing cell death [19]. We reasoned that this translocation is a mechanism to avoid being killed in the phagolysosome. This cytosolic replication of Mtb in hLECs represents a fundamental difference when compared to macrophages, where cytosolic translocation is associated with cell death [49] and extensive Mtb replication in dead cells [48]. Thus, the reduced ability of the PDIM strain to localise in the cytosol would largely explain why PDIMs are crucial for replication in hLECs, whereas it is much less crucial (or not at all) in macrophages. However, there was still 21% of PDIM-negative Mtb present in the cytosol, yet this seems not to contribute towards increasing the intracellular burden. Therefore, there are likely further mechanisms by which PDIM indirectly or directly affects replication in hLECs. One possible mechanism could be related to the reported higher permeability of the Mtb cell wall in PDIM-negative strains of Mtb [29]. It is feasible that PDIM-less Mtb is more sensitive to an antibacterial cytosolic component than Mtb WT such as nitric oxide from endothelial nitric oxide synthase, which we previously found to target cytosolic Mtb [19].

Extrapulmonary TB accounts for 25% of all cases and infection of the lymph nodes represents a key stage in TB dissemination. Therefore, lack of growth in hLECs by PDIM-deficient Mtb could contribute to the many reasons why there is discordance between the reports of only a minor effect of PDIM deficiency in macrophages in cellulo and PDIM being essential for virulence in vivo. In addition, this suggests that targeting PDIM synthesis pharmacologically could significantly reduce the lymphatic reservoir of Mtb in vivo, subsequently reducing both dissemination and reactivation of Mtb. Supporting this, one of the two known mechanisms of action of pyrazinamide (a first-line anti-tubercular drug) targets PDIM synthesis [50], but the effects of this drug on the lymphatic pool of Mtb specifically due to targeting PDIM synthesis is unknown.

With the increasing recognition of PDIM as a key factor for Mtb–host interactions, it is essential to consider the PDIM status of strains used for infection. Furthermore, Mtb is likely to exist as a heterogenous population in respect to PDIM/ESX-1 expression and secretion levels, even when prepared as a clonal population. These factors may explain some of the many discrepancies regarding intracellular trafficking, replication, induction of host cell death, type of host cell death and intracellular/extracellular signalling that are present in the literature and should be considered.

## Conclusions

We determined that there was at least a 50% decrease in the extent of phagosome damage when PDIMs were absent from Mtb in hLECs after 5 + 2 h of infection. The reduced phagosome damage correlated with an increased presence of Mtb in phagolysosomes. We also found that a lack of PDIM led to reduced recruitment of xenophagy markers, most likely due to the lack of phagosome membrane damage and therefore, reduced access for xenophagy receptors to Mtb and/or the cytosolic surveillance pathway to Mtb-derived nucleic acids. Ultrastructural analysis confirmed that Mtb lacking PDIM was much less frequently localised in the cytosol in hLECs after 5 + 24 h of infection. Finally, we determined that the intracellular burden of Mtb lacking PDIM is severely reduced in hLECs. This work highlights PDIM as a crucial virulence factor for Mtb in hLECs and suggests that pharmacological targeting of PDIM synthesis may lead to a reduced reservoir of Mtb in the lymphatic system in humans. For the wider tuberculosis field, this work further emphasises the importance of ensuring that cultured Mtb stocks are PDIM-positive, because PDIM is frequently lost during in vitro culturing [6] and this can have multiple deleterious consequences in cellulo.

## Methods

### Eukaryotic cell culture

Primary hLECs (ScienCell Research Laboratories) were cultured according to the supplier’s instructions up to passage 5. hLECs were seeded at a density of 5000 cells per cm^2^ into T-75 flasks that had been coated with fibronectin (Sigma-Aldrich) overnight at 37 °C. hLECs were incubated at 37 °C with 5% CO_2_ overnight in endothelial cell medium (ECM) (ScienCell Research Laboratories) supplemented with 5% (v/v) foetal bovine serum (ScienCell Research Laboratories) and 1% (v/v) endothelial cell growth supplement (ScienCell Research Laboratories). The cells were then washed with phosphate buffered saline (PBS) and the culture medium replaced. The cells were grown until 90% confluent, detached from the flask using 2.5 mL 0.25% Trypsin-EDTA (Gibco) and used for experimentation (or passaged into new flasks). For confocal microscopy, 10,000 hLECs in 300 μL with complete ECM were seeded onto fibronectin-treated 1.5-thickness 10-mm-diameter glass coverslips (Glaswarenfabrik Karl Hecht) placed into each well of 24-well plates treated with tissue culture. For electron microscopy, 300,000 hLECs were seeded in 5 mL with complete ECM in fibronectin-treated T-25 flasks.

### Bacterial strains and culture

Mtb strains used in this study were kindly provided by C. Asterie-Dequeker as used in [10]. A detailed description of these strains including the method used to knock out and complement the *ppsE* gene, as well as fluorescently label the bacteria using an integrating GFP tag, is provided in [10]. In this study, we refer to the Mtb-GFP WT strain as Mtb WT, the Mtb-GFP PMM100 strain as Mtb ΔPDIM and the Mtb-GFP PMM100::pMVE strain as Mtb ΔPDIM::PDIM. Culture of the mycobacteria was in Middlebrook’s 7H9 broth medium (Sigma-Aldrich) supplemented with 0.05% (v/v) glycerol (Fisher Chemical), 10% (v/v) Middlebrook albumin-dextrose-catalase (BD Biosciences) and 0.05% (v/v) Tween80 (Sigma-Aldrich). Cultures of 10 mL were incubated at 37 °C with rotation in 50-mL Falcon tubes.

### Infection of hLECs with Mtb

Mtb was grown to mid-exponential phase and pelleted at 2000 *g* in a benchtop centrifuge for 5 min at room temperature (RT). A pellet was washed once with 10 mL PBS and once with 10 mL ECM, re-pelleting after each wash. Five sterile 2.5–3.5 mm glass balls (VWR) were added to the pellet and vigorously shaken for 1 min to break up clumps of bacteria. Then, 10 mL of ECM was used to rinse the sides of the Falcon tube and resuspend the bacteria. Next, a slow spin at 300 *g* for 5 minutes was performed and the top 8 mL of the suspension was carefully transferred to a new Falcon tube. OD_600_ was measured and the mixture diluted in ECM to achieve a MOI of ten bacteria to one hLEC (assuming a cell suspension of OD 0.1 contains 10^7^ mycobacteria per mL; data not shown) in a total volume of 300 μL of ECM per well in a 24-well plate or 2 mL of ECM per T-25 flask. After 5 h of infection (at 37 °C with 5% CO_2_), the cells were washed twice with PBS to remove extracellular bacteria and then 1 mL (for 24-well plates) or 5 mL (for T-25 flasks) of complete ECM was added and incubated for 2, 24, 48 and 72 hours.

### Antibodies

The following primary antibodies were used in this study: goat anti-Galectin 8 (R&D Systems, AF1305; AB_2137229; 1:250 dilution), rabbit anti-CtsD (provided by Andrej Hasilik; 1:100 dilution), mouse anti-ubiquitin FK2 (Enzo, BML-PW8810; batch 08101527; AB_10541840; 1:100 dilution), rabbit anti-p62 (GeneTex, GTX111393; batch 40450; AB_10723101; 1:500 dilution), rabbit anti-NDP52 (provided by J. Kendrick-Jones; 1:250 dilution), rabbit andti-IRF-1 (Cell Signalling, 8478 lot 2; 1;250 dilution) and rabbit anti-p65 (Abcam, ab16502; batch GR3182233; 1:250 dilution). All were incubated for 1 h at RT at the dilution shown. The following secondary antibodies were used in this study at 1:800 dilution: goat anti-rabbit Alexa Fluor-568 (Thermo Fisher; A11036; Batch 1704462 AB_143011), goat anti-mouse Alexa Fluor-633 (Thermo Fisher; A21052; batch 1712097; AB_141459) and bovine anti-goat Cy3 (Jackson ImmunoResearch; 805-165-180; AB_2340880).

### Indirect immunofluorescence

At the required time point, hLECs infected with Mtb on glass coverslips were fixed by removing the culture medium, washing once with 1 mL of PBS and then adding PBS containing 3% (v/v) methanol-free paraformaldehyde (Electron Microscopy Sciences) overnight at 4 °C. The samples were then quenched with 1 mL of 50 mM NH_4_Cl (Sigma-Aldrich) in PBS for 15 mins at RT and then permeabilised with 500 μL 0.05% (w/v) saponin (Sigma-Aldrich) and 1% (w/v) bovine serum albumin (BSA; Sigma-Aldrich) in PBS for 10 mins at RT. After one PBS wash for 5 mins at RT, the coverslips were transferred onto a sheet of parafilm attached to the bench. Before the coverslips could dry out, 50 μl of diluted primary antibody (in PBS containing 0.05% saponin and 1% BSA) was added onto the coverslip and then incubated in the dark with a moist tissue for 1 h at RT. Next, the coverslips were transferred back into the wells of a 24-well plate and washed three times with 1 mL of PBS for 5 min each. The secondary antibody was added in the same way as the primary. After two more PBS washes, DNA was stained using 1 mL of 300 nM DAPI (Life Technologies) in PBS for 10 mins at RT. The coverslips were washed once more and finally were mounted onto glass slides using 8 μL DAKO mounting medium (DAKO Cytomation).

### Fluorescence imaging and analysis

We used a Leica SP5 laser scanning confocal microscope with an acousto-optical beam splitter (Leica Microsystems) equipped with HC PL APO CS2 63×, 1.4 NA oil objective, UV laser (excitation at 405 nm), argon laser (488 nm), diode-pumped solid-state laser (561 nm), HeNe laser (633 nm), one photomultiplier tube detector and two HyD detectors for all imaging of fixed samples, with Leica Type F (Leica Microsystems) immersion oil. Image acquisition used the proprietary Leica LAS AF software (Leica Microsystems) with the following settings: argon laser set to 20%, scanning mode xyz, sequential acquisition, pixel resolution of 1024 × 1024, scanner frequency 200 Hz, line averaging 4, zoom 1, 8-bit acquisition. All acquisition settings were kept constant between repeats. At least six random fields of view were analysed for each sample, with at least three independent repetitions performed (unless stated otherwise).

Leica SP5 files (lif) were opened using FIJI, a distribution of ImageJ (National Institutes of Health). To measure the intracellular replication of Mtb (i.e. the GFP signal per hLEC), a cell was outlined using the ‘line’ tool to create a region of interest and the GFP channel was duplicated. Anything outside the region of interest was cleared, and the GFP channel was subjected to a consistent pixel threshold of 50, thus creating a binary image where each GFP pixel had a value of 255. The sum value of the GFP pixels is called RAWIntDen. Because each GFP pixel is 0.24 × 0.24 μm and has a value of 255, RAWIntDen can be converted to total GFP area per hLEC (μm^2^) using the following equation: (RAWIntDen × (0.24 × 0.24))/255.

To measure the association of a marker (e.g. Gal8) with the bacteria, we measured the mean intensity of all the pixels in the marker channel that overlap with the GFP from the bacteria channel. This was done by splitting the multi-channel image into individual channels and subjecting the GFP channel to a consistent pixel threshold of 50 to create a mask. The mask was subjected to the ‘fill holes’, ‘dilate’ (1 pixel) and ‘erode’ (1 pixel) tools in that order to create a good representation of the true outlines of the bacteria. Then the ‘analyse particles’ function of FIJI was used and measurements were re-directed to the marker channel.

### Ultrastructural analysis

After 5 + 24 h of infection, hLECs infected with Mtb in T-25 flasks were fixed by addition of warm 2% glutaraldehyde in 200 mM HEPES pH 7.4 directly to the culture medium at a 1:1 volume ratio. After 5 min, this was replaced with 5 mL of 1% glutaraldehyde in 200 mM HEPES and kept at pH 7.4 overnight at 4 °C. Subsequently, cells were gently scraped and embedded in 1% low-melting-point agarose (Thermo Fisher Scientific). Blocks were post-fixed with 2% osmium tetroxide solution (Electron Microscopy Sciences) containing 1.5% potassium ferricyanide for 2 h on ice, then stained with 1% tannin for 30 min and with 2% aqueous uranyl acetate (Electron Microscopy Sciences) for 2 h at RT. Next, cells were dehydrated at RT starting with a graded ethanol series (70%, 80%, 90%, 95% and 100%), ending with propylene oxide, followed by gradual infiltration with Spurr's resin (Polysciences) over 2 days. Ultrathin sections (~70 nm) were cut using an ultramicrotome Ultracut EM UCT (Leica Microsystems) and a diamond knife (Diatome), and contrasted with 0.2% lead citrate for 15 s. Sections were analysed with a JEM-1400 TEM (JEOL). Images were taken with a TemCam-F216 camera and EM-MENU software (TVIPS).

At least 32 different infected cells per sample were imaged by systematic random sampling. For subcellular localisation analysis, cytosolic, phagosomal/phagolysosomal or autophagic bacteria were classified as described in the legend to Fig. 3. Their relative proportions were determined by counting cross-points of the stereological test grid over bacteria. Proportions were calculated from total counts per sample.

### Data and statistical analysis

Results are expressed as means from three independent experiments (unless otherwise stated) ± standard error of the mean. Data were plotted and statistical analyses were performed using Graphpad Prism 6 (GraphPad Software Inc). Means between three or more groups were tested for significant differences using one-way ANOVA with Tukey’s post-test.

## Abbreviations

ANOVA: Analysis of variance
BSA: Bovine serum albumin
CtsD: Cathepsin D
DAPI: 4',6-diamidino-2-phenylindole
ECM: Endothelial cell medium
ESAT-6: Early secretory antigenic target of 6 kDa
ESX-1: ESAT-6 secretion system
Gal8: Galectin 8
GFP: Green fluorescent protein
hLEC: Human lymphatic endothelial cell
hpi: Hours post-infection
LC3: Microtubule-associated protein 1A/1B light chain 3
MOI: Multiplicity of infection
Mtb: *Mycobacterium tuberculosis*
NA: numerical aperture
NDP52: antigen nuclear dot 52 kDa protein
ns: Not significant
p62: Sequestosome 1/nucleoporin 62
PBS: Phosphate-buffered saline
PDIM: Phthiocerol dimycocerosate
RT: Room temperature
TB: Tuberculosis
TEM: Transmission electron microscopy
Ub: Ubiquitin
WT: Wild type
